# Macronutrient content and fatty acid composition and their positional distribution in human breast milk from Zhejiang Province, China in different lactation periods

**DOI:** 10.1002/fsn3.2626

**Published:** 2021-10-20

**Authors:** Guanghua He, Guipu Li, Yanxi Jiang, Jiacai Hua, Xiaojun Chu, Lina Xiong, Jinyan Gong, Gongnian Xiao, Xingqian Ye

**Affiliations:** ^1^ Department of Food Science and Nutrition Zhejiang University Hangzhou China; ^2^ School of Biological and Chemical Engineering Zhejiang University of Science and Technology Hangzhou China; ^3^ Beingmate (Hangzhou) Food Research Institute Co., Ltd Hangzhou China

**Keywords:** fatty acid composition, human breast milk, infant formulas, lipid profile, positional distribution

## Abstract

Lactational changes in macronutrient content, lipid profile, fatty acid composition, and positional distribution of human breast milk were investigated in this study. A total of 378 milk samples of six different lactation periods, including 0‒5, 6‒14, 15‒30, 31‒90, 91‒180, and 181‒360 days, were collected cross‐sectionally from healthy lactating women in Zhejiang, China. As lactation progressed from 0‒5 to 15‒30 days, the lipid content and the percentages of C10:0, C12:0, C14:0, C18:2*n*‐6, and C18:3*n*‐3 increased significantly, while the protein concentration and the proportions of phospholipids, cholesterols, C16:0, C18:1*n*‐9, C24:1*n*‐9, C20:4*n*‐6, C22:4*n*‐6, C22:5*n*‐3, and C22:6*n*‐3 decreased notably. When lactation was further extended to 181‒360 days, the protein content continued to decrease, and the percentages of C12:0 and C14:0 continued to increase, whereas the levels of other tested nutrients remained stable. Although the triacylglycerol positional distributions of some fatty acids underwent significant lactational variations, C14:0, C16:0, C24:1*n*‐9, C22:4*n*‐6, C22:5*n*‐3, and C22:6*n*‐3 were located mainly at the *sn*‐2 position, while C18:1*n*‐9, C18:2*n*‐6, and C18:3*n*‐3 were primarily distributed at the *sn*‐1,3 positions. Compared with human breast milk reported in Western countries, samples in our study demonstrated higher percentages of C18:2*n*‐6, C18:3*n*‐3, C20:4*n*‐6, and C22:6*n*‐3, but lower proportions of C12:0, C14:0, and C18:1*n*‐9. The results from this study indicated a nutritional composition different from that of the Western countries and may provide useful data for the development of infant formulas closer to Chinese breast milk in terms of the fatty acid composition and its specified positional distribution on triglyceride structure.

## Graphical abstract

Human milk from Zhejiang Province of China demonstrated significant lactational variations in fatty acid composition. As lactation progressed from 0‒5 to 181‒360 days, C12:0 and C14:0 showed a notable rising tendency, C18:2*n*‐6 and C18:3*n*‐3 increased significantly first and then remained constant, while C16:0, C18:1*n*‐9, C20:4*n*‐6, C22:5*n*‐3 and C22:6*n*‐3 decreased first and then maintained consistently. Regardless of lactational variations, C14:0, C16:0, C22:5*n*‐3 and C22:6*n*‐3 were located mainly at the s*n*‐2 position of triacylglycerol, while C18:1*n*‐9, C18:2*n*‐6 and C18:3*n*‐3 were primarily distributed at the sn‐1,3 positions.

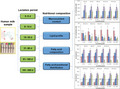

## 1 INTRODUCTION

Human breast milk from healthy and well‐nourished mothers is universally recognized as the optimal natural food for newborn infants (Victora et al., 2016). It provides perfectly balanced nutrition to meet the needs of the growing infant in the first several months after birth (Stam et al., 2013). The World Health Organization (WHO) and American Academy of Pediatrics (AAP) recommend that infants should be exclusively breastfed for the first 6 months of life and continuously breastfed by introducing appropriate complementary foods from 6 months through 1‒2 years (American Academy of Pediatrics, 2012; Fewtrell et al., 2007; World Health Organization, 2001). Unfortunately, human milk is not always available for certain reasons; thus, infant formula that mimics human milk is regarded as the best alternative (Wei et al., 2019).

The contents of macronutrients, including protein, lipids, and lactose, in human breast milk, are crucial and commonly used to estimate the nutrient requirements for infants (Zhu et al., 2017). Among the macronutrients, human milk lipids contribute a major proportion (45%‒55%) of the total energy supply and provide complex lipids, fatty acids, and bioactive components that are essential for rapid development and appropriate growth of infants (Koletzko, 2016). Therefore, it is critical to understand human milk macronutrient content and lipid composition to guide infant feeding and formula development.

Human breast milk is special in lipid profile, fatty acid composition, and positional distribution compared with other mammalian milk (bovine milk, goat milk, etc.) and vegetable oil, which are commonly used as the lipid sources of infant formula (Demmelmair & Koletzko, 2018; Koletzko, 2016). In the case of the lipid profile, human milk contains triacylglycerols (TAGs), phospholipids (PLs), cholesterols (CHOLs), monoacylglycerols (MAGs), diacylglycerols (DAGs), and free fatty acids (FFAs), among which TAGs account for approximately 98% of the total lipids and contribute the major source of fatty acids (Koletzko, 2016; Liu et al., 2018), whereas PLs and CHOLs are minor in content (<1%) but have beneficial effects on the rapid development of the brain in infancy (Cartocci et al., 2017; Cilla et al., 2016; Dietschy & Turley, 2004). Regarding fatty acid composition and positional distribution, human milk lipids provide 35%‒55% saturated fatty acids (SFAs), 25%‒40% monounsaturated fatty acids (MUFAs), and 10%‒25% polyunsaturated fatty acids (PUFAs; Floris et al., 2020; Wei et al., 2019). These fatty acids are selectively esterified in a regular arrangement at three stereospecifically numbered (*sn*) positions of the TAG glycerol skeleton. Palmitic acid (PA, C16:0), the most abundant SFA, is located mainly at the *sn*‐2 position of the TAG, whereas unsaturated fatty acids, including oleic acid (OA, C18:1*n*‐9), linoleic acid (LA, C18:2*n*‐6), and α‐linolenic acid (ALA, C18:3*n*‐3), are preferentially distributed at the outer *sn*‐1,3 positions (Bar‐Yoseph et al., 2013; Innis, 2011). This specific fatty acid positional distribution has been shown to be beneficial for fatty acid and mineral absorption, bone health, gut microbial development, and infant comfort (Jiang et al., 2018; Litmanovitz et al., 2013; López‐López et al., 2001; Savino et al., 2006). Hence, not only the lipid profile and fatty acid composition but also the positional distribution of fatty acids on the triglyceride structure of human milk should be considered when developing infant formula.

To date, numerous studies on human breast milk composition have been carried out in western regions and have revealed that the macronutrient content, lipid profile, and fatty acid composition of human milk vary with lactation periods and geographical regions due to diverse dietary patterns and genetic backgrounds (Bravi et al., 2016; Gidrewicz & Fenton, 2014; Innis, 2014; Sosa‐Castillo et al., 2017). To our knowledge, several studies in this field have been conducted in China, but only a few of these studies were involved in fatty acid positional distribution (Deng et al., 2018; Qi et al., 2018; Wu et al., 2019). In addition, most of the studies merely focused on milk samples within a lactation period of fewer than three months, which may not provide sufficient data support for the development of localized infant formulas at different stages (0–6 months and 6–12 months). Geographically representative breast milk from healthy, well‐nourished mothers should be considered as the golden reference in the development of localized infant formulas. Zhejiang, one of the most developed provinces in China with typically healthy dietary patterns and good improvements in infant growth and development (weight, height, head circumference, nutritional status, etc.), is an ideal area for the study of Chinese human milk.

In the present study, we investigated the macronutrient content, lipid profile, fatty acid composition, and positional distribution of human breast milk in six different lactation periods (0‒5, 6‒14, 15‒30, 31‒90, 91‒180, and 181‒360 days) from healthy lactating women in Zhejiang, and compared with those of breast milk from Western countries. Our aim was to elucidate the characteristics of the lipid composition and structure of Chinese breast milk and to explain the differences with those of Western breast milk. The results provide a broad overview of the fatty acid composition events underlying the lactation periods changes and explore the valuable specified positional distribution of fatty acids on triglyceride structure of human milk that could be used to develop commercial infant formulas more suitable for Chinese babies.

## 2 MATERIALS AND METHODS

### 2.1 Subjects and milk sample collection

Three hundred and seventy‐eight lactating women from Zhejiang Province of China who met the inclusion criteria were recruited for the study. The inclusion criteria were as follows: (1) Healthy breastfeeding mothers of full‐term and singleton delivery babies; (2) no smoking and drinking; (3) no severe nutritional diseases (iron‐deficiency anemia, marasmus malnutrition, kwashiorkor, etc.) and malignant consumptive diseases (malignant tumor, pulmonary tuberculosis, etc.); (4) no chronic diseases of diabetes and hypertension; and (5) no nutritional supplements were taken recently. The basic characteristics of the participants, including childbirth age, gestational period, pregestation, and predelivery body mass indices (BMI), as well as birth weight and length of respective babies, were collected at the time of enrollment by a self‐administered questionnaire. All participants received detailed information about the study and provided written informed consent. The study protocol was approved by the Ethics Committee of the College of Biosystem Engineering and Food Science, Zhejiang University.

Each participant was asked to provide one milk sample between 9:00 am and 12:00 am. Both sides of the mammary gland were evacuated completely with an electric breast pump, and the milk was carefully mixed. A portion of 20 ml of whole breast milk was taken and subpacked into four 5‐ml frozen tubes, and then immediately stored in a low‐temperature refrigerator at −80°C until analysis. The remaining milk was fed to the infants. Among the four frozen tubes, one is used for macronutrient content analysis, two were used for lipid and fatty acid composition determination, and the remaining one was used as a backup sample.

### 2.2 Macronutrient content analysis

Macronutrient content, including lipids, protein, and lactose, of human breast milk were determined by injecting an aliquot of a 2‐ml milk sample into a mid‐infrared human milk analyzer (HMA, Miris AB) according to the manufacturer's instructions (Zhu et al., 2017). Before analysis, the milk samples were thawed in a water bath at 40°C and homogenized by an ultrasonic oscillatory mixing machine. A daily calibration check and cleaning steps for every 10 analyses were performed using the calibration solution and cleaning solution, respectively. The accuracy (average recoveries, *n* = 6) and precision (relative standard deviations (RSD), *n* = 6) of the method were 88.46%‒116.94% and 0.37%‒1.86%, respectively.

### 2.3 Total lipid extraction

Total lipids from the breast milk were extracted using the Association of Official Analytical Chemists (AOAC) official method 989.05 with some modification (Barbano et al., 1988). In brief, 5 ml of thawed and homogenized breast milk and 1 ml of ammonia water were placed in a 50‐ml centrifuge tube, mixed thoroughly, and incubated in a water bath at 65°C for 20 min. After the mixture was cooled to room temperature, 5 ml of absolute ethanol was added and mixed for 1 min. Then, 12.5 ml of anhydrous ether and 12.5 ml of petroleum ether were added and mixed for 3 min to extract the lipids. The mixture was centrifuged for 15 min at 1500*g*, and the clear supernatant was collected and dried with blowing nitrogen to obtain the milk lipid extract. Portions of 10, 20, and 30 mg of the lipid extract were taken to determine the lipid profile, total fatty acid composition, and *sn*‐2 fatty acid composition, respectively.

### 2.4 Lipid analysis

The lipid composition was analyzed using an Iatroscan thin‐layer chromatography with flame ionization detector (TLC‐FID) analyzer (Iatron Laboratories Inc.; Li et al., 2010). Briefly, 10 mg of human milk lipid extract, which had been dissolved in 1 ml of chloroform, was spotted on the starting point of the chromarods as 2 µl using an autospotter. After spotting, the rods were developed in developing tanks with a petroleum ether‐ethyl ether‐acetic acid (60:15:0.1, v/v/v) solvent system for 25 min and a petroleum ether‐ethyl ether (56:4, v/v) solvent system for 30 min successively. Each time after development, the rods were placed in an oven (53°C) for 3 min to evaporate the residual solvents. Then, the chromarods were scanned with an Iatroscan MK‐6s TLC‐FID Analyzer. The air and hydrogen flow rates were 2000 ml/min and 160 ml/min, respectively, and the scan speed was set at 30 s/scan. The compositions of lipid classes were expressed in weight percentages (wt %) of the total lipids according to their peak areas, which were recorded and processed by Chromstar software. Glyceryl tripalmitate, lecithin, cholesteryl palmitate, cholesterol, glyceryl 1,2‐dipalmitate, glyceryl 1,3‐dipalmitate, and palmitic acid (Sigma–Aldrich) were employed as authentic standards for the quantitative analysis of TAGs, PLs, cholesterol esters (CEs), free cholesterols (FCHOLs), 1,2‐DAGs, 1,3‐DAGs, and FFAs. The accuracy (average recoveries, *n* = 6) and precision (RSD, *n* = 6) of the method were 92.67%‒104.05% and 0.24‒7.76%, respectively.

### 2.5 Fatty acid methylation

Milk lipid extract (20 mg) was dissolved in 0.7 ml of potassium hydroxide‐methanol solution (1 mol/L) and incubated in a water bath at 60°C for 3 min with shaking. Then, 1 ml of boron trifluoride‐methanol solution (14%, w/v) was added and incubated in a water bath at 50°C for 15 min. After the mixture was cooled to room temperature, 2 ml of saturated NaCl solution and 1 ml of *n*‐hexane were added and mixed thoroughly and then centrifuged at 2200*g* for 5 min. The supernatant containing fatty acid methyl esters (FAMEs) was collected and subjected to gas chromatographic (GC) analysis.

### 2.6 2‐Monoacylglycerol (2‐MAG) and its FAME preparation

Human milk lipids were hydrolyzed to 2‐MAG by means of the method described by Luddy et al. (1964) with some modification. Lipid extract (30 mg), Tris‐HCl buffer (1 mol/L, 2 ml), sodium cholate (1 g/L, 0.5 ml), and calcium chloride (2.2%, 0.2 ml) were added to a 10 ml test tube and incubated in a water bath at 40°C for 1 min with shaking. Porcine pancreatic lipase (30 mg, type II, Sigma–Aldrich) was then added, and the mixture was mixed well and incubated in a water bath at 40°C for another 3 min. After the mixture was cooled, the HCl solution (6 mol/L, 1 ml) and diethyl ether (2 ml) were added and the solution was subjected to centrifugation at 4000 r/min for 5 min. The obtained clear upper phase containing hydrolytic product was collected and evaporated to dryness under nitrogen gas. The residual‐dried hydrolytic product was redissolved in 1 ml of *n*‐hexane, and the 2‐MAG in the hydrolytic product was separated and purified by an NH_2_ solid‐phase extraction cartridge (500 mg, 6 ml; Anpel). Acetone (5 ml) and *n*‐hexane (5 ml) were used to activate the cartridge. Then, the hydrolytic product was loaded on the cartridge and eluted by 5 ml of acetone/*n*‐hexane (15:85, v/v) and 5 ml of acetone/*n*‐hexane (50:50, v/v) successively. The eluate of acetone/*n*‐hexane (50:50, v/v) containing 2‐MAG was collected and dried by nitrogen gas blowing and then subjected to the same methylation procedure as above to produce FAMEs.

### 2.7 GC analysis

The FAME was analyzed using an Agilent GC 7890A (Agilent Corporation) equipped with a FID and a 100 m × 0.25 mm × 0.20 μm capillary column (Supelco 2560, Sigma–Aldrich). The injector and detector temperatures were 240°C and 250°C, respectively. Nitrogen was used as the carrier gas with a flow rate of 1.0 ml/min, and the split ratio was 1:10. The column temperature program was as follows: 60°C held for 2 min, followed by an increase of 15°C/min to 100°C, and subsequently elevated to 230°C at the rate of 3°C/min and maintained for 18 min. Fatty acids were identified by the comparison of retention time with a FAME standard mixture (GLC‐746, Nu‐Chek Prep). The compositions of fatty acids were expressed as weight percentages (wt %) of total fatty acids according to their peak areas. The accuracy (average recoveries, *n* = 6) and precision (RSD, *n* = 6) of the method were 94.18%‒109.37% and 1.28%‒6.45%, respectively.

### 2.8 Fatty acid positional distribution calculation

The fatty acid positional distribution was calculated as the relative percentage of a specific fatty acid at the *sn*‐2 position compared with the total amount of this fatty acid using the following equation: *sn*‐2/total, % = percentage in total *sn*‐2 fatty acids/(percentage in total fatty acids × 3) × 100.

### 2.9 Statistical analysis

All determinations were made in duplicate, and the results were reported as the mean ± standard deviations (SD). Before statistical analyses, data were checked for normal distribution and variance homogeneity using the Shapiro–Wilk and Levene tests, respectively. If the data were normally distributed, one‐way analysis of variance (ANOVA) was adopted to compare the nutritional composition of human milk in different lactation periods, and the Tukey–Kramer multiple comparison test was applied to test for differences between means at the 5% significance level (*p* < .05). Otherwise, a nonparametric Kruskal–Wallis test was used. One‐way analysis of covariance (ANCOVA) was used to compare the nutritional composition of human milk in different lactation periods with adjustment for childbirth age, gestational period, pregestation and predelivery BMI, and Bonferroni multiple comparison test was employed to test for differences between means at the 5% significance level (*p* < .05). All analyses were conducted using SPSS 20.0 (SPSS Inc.).

## 3 RESULTS

### 3.1 Basic characteristics

A total of 378 milk samples in 6 different lactation periods, including 0‒5 days (*n* = 60), 6‒14 days (*n* = 58), 15‒30 days (*n* = 55), 31‒90 days (*n* = 70), 91‒180 days (*n* = 70), and 181‒360 days (*n* = 65), were collected cross‐sectionally from 378 healthy lactating women. The basic characteristics of mothers and respective babies are shown in Table 1. Childbirth age (27.5‒28.9 years), gestational period (38.7‒39.4 weeks), pregestation (20.8‒21.8 kg/m^2^), and predelivery BMI (26.7‒27.9 kg/m^2^) of sampled mothers, as well as birth weight (3.43‒3.67 kg) and length (50.0‒51.0 cm) of respective babies, were similar among different lactation periods.

**TABLE 1:** Basic characteristics of mothers and respective babies corresponding to 378 breast milk samples with different lactation periods

Basic characteristics		Lactation period						*p* value
		0–5 days (*n* = 60)	6–14 days (*n* = 58)	15–30 days (*n* = 55)	31–90 days (*n* = 70)	91–180 days (*n* = 70)	181–360 days (*n* = 65)	
Mothers	Childbirth age (years)	28.6 ± 3.5	27.5 ± 4.0	27.6 ± 4.2	28.9 ± 4.8	27.6 ± 3.9	28.9 ± 4.3	.291
	Gestational period (weeks)	39.0 ± 1.3	39.3 ± 1.9	38.7 ± 1.8	39.4 ± 1.1	39.2 ± 1.1	39.1 ± 1.1	.126
	BMI (kg/m^2^), pregestation	21.1 ± 2.3	21.5 ± 4.0	21.8 ± 2.8	20.9 ± 3.7	20.8 ± 2.1	21.7 ± 3.5	.487
	BMI (kg/m^2^), predelivery	26.7 ± 2.8	27.3 ± 4.5	27.9 ± 3.5	27.7 ± 3.8	26.9 ± 3.5	27.4 ± 3.5	.124
Babies	Birth weight (kg)	3.67 ± 0.93	3.49 ± 0.42	3.65 ± 0.84	3.43 ± 0.71	3.63 ± 0.99	3.48 ± 0.72	.333
	Birth length (cm)	51.0 ± 2.1	50.4 ± 1.8	50.0 ± 2.8	50.1 ± 2.3	50.3 ± 2.3	50.5 ± 1.8	.503

Note

Results are presented as mean ± SD.

### 3.2 Macronutrient content

The macronutrient content of human breast milk from different lactation periods is listed in Table 2. The lipid content of breast milk increased dramatically from 2.81 g/100 ml at lactation 0‒5 days to 4.05 g/100 ml at lactation 31‒90 days and then remained stable thereafter, while the protein content decreased significantly during the whole lactation period, from 2.19 g/100 ml at lactation 0‒5 days to 1.07 g/100 ml at lactation 181‒360 days. Lactose content (6.63‒6.97 g/100 ml) was consistent among different lactation times.

**TABLE 2:** Macronutrient contents (g/100 ml) of human breast milk from different lactation periods

Macronutrients	Lactation period						*p* value
	0‒5 days	6–14 days	15–30 days	31–90 days	91–180 days	181–360 days	
Lipid	2.81 ± 0.74c	3.52 ± 0.65b	3.81 ± 0.87ab	4.05 ± 1.28a	3.89 ± 1.37ab	3.98 ± 1.16a	<.001
Protein	2.19 ± 0.44a	1.77 ± 0.33b	1.45 ± 0.38c	1.21 ± 0.27d	1.14 ± 0.22de	1.07 ± 0.19e	<.001
Lactose	6.63 ± 0.54	6.86 ± 0.74	6.96 ± 0.51	6.91 ± 0.65	6.97 ± 0.67	6.88 ± 0.45	.102

Note

Results are presented as mean ± SD. Values within the same row not sharing a common letter are significantly different (*p* < .05).

### 3.3 Lipid profile

Table 3 shows the lipid profile of human breast milk from different lactation periods. The percentages of TAGs, PLs, CEs, FCHOLs, MAGs, DAGs, and FFAs in total lipids were 97.15%‒97.77%, 0.68%‒1.32%, 0.27%‒0.49%, 0.19%‒0.32%, 0.20%‒0.30%, 0.13%‒0.23%, 0.14%‒0.22%, and 0.24%‒0.40%, respectively. All lipid classes underwent significant variations among breast milk of lactation periods of 0‒5, 6‒14, and 15‒30 days. Samples obtained at lactation 0‒5 days demonstrated significantly higher levels of PLs, CEs, and FCHOLs but lower levels of TAGs, MAGs, 1,2‐DAGs, 1,3‐DAGs, and FFAs than those obtained at lactation 15‒30 days. No significant changes were found in the percentages of all lipid classes among milk samples of lactation periods of 15‒30, 31‒90, 91‒180, and 181‒360 days.

**TABLE 3:** Lipid profile (wt% of total lipids) of human breast milk from different lactation periods

Lipid class	Lactation period						*p* value
	0–5 days	6–14 days	15–30 days	31–90 days	91–180 days	181–360 days	
TAGs	97.15 ± 0.32c	97.48 ± 0.31b	97.72 ± 0.29a	97.77 ± 0.31a	97.66 ± 0.30a	97.74 ± 0.28a	<.001
PLs	1.32 ± 0.21a	0.90 ± 0.18b	0.73 ± 0.13c	0.70 ± 0.16c	0.74 ± 0.18c	0.68 ± 0.15c	<.001
CEs	0.49 ± 0.12a	0.31 ± 0.11b	0.27 ± 0.10b	0.28 ± 0.12b	0.28 ± 0.10b	0.29 ± 0.09b	<.001
FCHOLs	0.32 ± 0.11a	0.25 ± 0.10b	0.19 ± 0.07c	0.19 ± 0.08c	0.21 ± 0.11c	0.20 ± 0.09c	<.001
MAGs	0.20 ± 0.08c	0.25 ± 0.09b	0.28 ± 0.09ab	0.29 ± 0.09a	0.30 ± 0.10a	0.28 ± 0.09ab	<.001
1,2‐DAGs	0.13 ± 0.05b	0.22 ± 0.07a	0.22 ± 0.06a	0.21 ± 0.07a	0.21 ± 0.05a	0.23 ± 0.06a	<.001
1,3‐DAGs	0.14 ± 0.06c	0.21 ± 0.07a	0.22 ± 0.09a	0.18 ± 0.06b	0.19 ± 0.07ab	0.22 ± 0.07a	<.001
FFAs	0.24 ± 0.08b	0.39 ± 0.09a	0.37 ± 0.10a	0.37 ± 0.09a	0.40 ± 0.09a	0.38 ± 0.07a	<.001

Note

Results are presented as mean ± SD. Values within the same row not sharing a common letter are significantly different (*p* < .05).

Abbreviations: CE, cholesterol ester; DAG, diacylglycerol; FCHOL, free cholesterol; FFA, free fatty acid; MAG, monoacylglycerol; PL, phospholipid; TAG, triacylglycerol.

### 3.4 Fatty acid composition

Table 4 shows the fatty acid composition of human breast milk from different lactation periods. A total of 29 fatty acids, including 11 SFAs, 7 MUFAs, and 11 PUFAs, were identified and determined. Total median‐chain SFAs (MCSFAs, C8:0‒C14:0), SFAs, MUFAs, *n*‐6 PUFAs, *n*‐3 PUFAs, and PUFAs, which varied significantly among different lactation breast milk samples, accounted for 6.04%‒10.45%, 35.78%‒39.05%, 35.58%‒40.70%, 20.62%‒23.79%, 2.12%‒2.46%, and 23.08%‒26.15% of the total fatty acids, respectively. Samples of lactation 0‒5 days demonstrated significantly lower percentages of total MCSFAs, SFAs, *n*‐6 PUFAs, and PUFAs but higher proportions of total MUFAs than those of lactation over 31 days.

**TABLE 4:** Fatty acid composition (wt% of total fatty acids) of human breast milk from different lactation periods

Fatty acids	Lactation period						*p* value
	0–5 days	6–14 days	15–30 days	31–90 days	91–180 days	181–360 days	
C8:0	0.12 ± 0.09c	0.14 ± 0.14bc	0.21 ± 0.14a	0.20 ± 0.13a	0.19 ± 0.06ab	0.19 ± 0.14ab	.002
C10:0	0.50 ± 0.41b	1.20 ± 0.57a	1.13 ± 0.45a	1.24 ± 0.43a	1.29 ± 0.36a	1.24 ± 0.36a	<.001
C12:0	2.37 ± 1.25e	3.66 ± 1.5 cd	3.27 ± 1.03d	3.91 ± 1.24bc	4.31 ± 1.35ab	4.61 ± 1.53a	<.001
C14:0	3.04 ± 1.35d	4.02 ± 1.12ab	3.39 ± 1.34 cd	3.78 ± 0.87bc	4.16 ± 1.12ab	4.41 ± 1.32a	<.001
Total MCSFAs	6.04 ± 2.37d	9.02 ± 2.94b	7.81 ± 2.67c	9.12 ± 2.29b	9.95 ± 2.6ab	10.45 ± 2.93a	<.001
C15:0	0.12 ± 0.04	0.11 ± 0.03	0.10 ± 0.05	0.10 ± 0.05	0.10 ± 0.06	0.11 ± 0.06	.081
C16:0 (PA)	22.53 ± 2.33a	22.73 ± 1.62a	21.29 ± 2.85b	20.70 ± 2.76b	20.37 ± 2.41b	20.53 ± 2.03b	<.001
C17:0	0.27 ± 0.04b	0.28 ± 0.06b	0.31 ± 0.10a	0.31 ± 0.07a	0.28 ± 0.06b	0.28 ± 0.04b	.001
C18:0	5.90 ± 1.00	6.04 ± 1.88	5.98 ± 1.02	6.15 ± 1.40	6.11 ± 1.10	6.06 ± 1.14	.927
C20:0	0.25 ± 0.07	0.27 ± 0.11	0.26 ± 0.06	0.27 ± 0.07	0.28 ± 0.09	0.27 ± 0.09	.515
C22:0	0.35 ± 0.14	0.39 ± 0.09	0.33 ± 0.16	0.32 ± 0.19	0.37 ± 0.19	0.34 ± 0.16	.296
C24:0	0.33 ± 0.13a	0.21 ± 0.11b	0.16 ± 0.10c	0.13 ± 0.08c	0.14 ± 0.09c	0.12 ± 0.09c	<.001
Total SFAs	35.78 ± 3.97d	39.05 ± 3.88a	36.45 ± 3.63 cd	37.08 ± 4.19bcd	37.61 ± 3.68abc	38.16 ± 4.46ab	.002
C14:1n−5	0.15 ± 0.10	0.14 ± 0.10	0.11 ± 0.07	0.15 ± 0.12	0.13 ± 0.09	0.11 ± 0.07	.067
C16:1n−7	1.49 ± 0.36b	1.64 ± 0.42ab	1.81 ± 0.51a	1.82 ± 0.53a	1.77 ± 0.47a	1.76 ± 0.53a	.005
C18:1 t	0.10 ± 0.04b	0.11 ± 0.04b	0.14 ± 0.07ab	0.12 ± 0.09ab	0.13 ± 0.09ab	0.15 ± 0.10a	.036
C18:1n−9 (OA)	37.55 ± 5.38a	34.03 ± 3.97b	33.90 ± 4.49b	33.87 ± 4.04b	33.46 ± 4.40b	32.88 ± 4.67b	<.001
C20:1n−9	0.85 ± 0.24a	0.59 ± 0.18b	0.56 ± 0.15b	0.49 ± 0.17c	0.48 ± 0.20c	0.47 ± 0.14c	<.001
C22:1n−9	0.29 ± 0.12a	0.17 ± 0.07b	0.17 ± 0.12bc	0.13 ± 0.10 cd	0.12 ± 0.09d	0.10 ± 0.05d	<.001
C24:1n−9	0.26 ± 0.11a	0.12 ± 0.06b	0.13 ± 0.08b	0.13 ± 0.07b	0.12 ± 0.06b	0.11 ± 0.05b	<.001
Total MUFAs	40.70 ± 5.51a	36.81 ± 4.17b	36.82 ± 4.66b	36.71 ± 4.03b	36.21 ± 4.49b	35.58 ± 4.65b	<.001
C18:2n−6 (LA)	17.06 ± 4.24c	18.86 ± 2.89b	21.87 ± 4.28a	21.42 ± 4.15a	21.40 ± 3.96a	21.75 ± 3.83a	<.001
C18:3n−6	0.19 ± 0.08b	0.16 ± 0.06b	0.29 ± 0.11a	0.29 ± 0.09a	0.31 ± 0.11a	0.30 ± 0.10a	<.001
C20:2n−6	1.11 ± 0.30a	0.55 ± 0.21b	0.43 ± 0.18c	0.42 ± 0.12c	0.40 ± 0.10c	0.44 ± 0.07c	<.001
C20:3n−6	0.46 ± 0.12a	0.35 ± 0.11b	0.28 ± 0.11c	0.26 ± 0.09 cd	0.23 ± 0.10de	0.21 ± 0.06e	<.001
C20:4n−6 (ARA)	1.08 ± 0.32a	0.84 ± 0.29b	0.63 ± 0.24c	0.61 ± 0.22c	0.58 ± 0.21c	0.60 ± 0.22c	<.001
C22:2n−6	0.23 ± 0.05a	0.17 ± 0.07b	0.13 ± 0.06c	0.11 ± 0.05 cd	0.10 ± 0.04de	0.09 ± 0.03e	<.001
C22:4n−6	0.50 ± 0.20a	0.28 ± 0.17b	0.16 ± 0.07c	0.17 ± 0.10c	0.18 ± 0.11c	0.16 ± 0.08c	<.001
Total *n*−6 PUFAs	20.62 ± 4.20b	21.20 ± 2.72b	23.79 ± 4.19a	23.28 ± 4.18a	23.19 ± 3.98a	23.54 ± 3.86a	<.001
C18:3n−3 (ALA)	1.10 ± 0.30c	1.32 ± 0.37b	1.57 ± 0.40a	1.55 ± 0.44a	1.59 ± 0.45a	1.44 ± 0.34ab	<.001
C20:5n−3	0.14 ± 0.08a	0.12 ± 0.08ab	0.11 ± 0.07b	0.11 ± 0.07b	0.10 ± 0.07b	0.09 ± 0.07b	.039
C22:5n−3 (DPA)	0.42 ± 0.18a	0.25 ± 0.12b	0.21 ± 0.11bc	0.20 ± 0.09bc	0.19 ± 0.10c	0.19 ± 0.07c	<.001
C22:6n−3 (DHA)	0.79 ± 0.30a	0.61 ± 0.28b	0.48 ± 0.22c	0.45 ± 0.19c	0.40 ± 0.16c	0.41 ± 0.16c	<.001
Total *n*−3 PUFAs	2.46 ± 0.51a	2.30 ± 0.58ab	2.36 ± 0.33a	2.30 ± 0.50ab	2.29 ± 0.58ab	2.12 ± 0.42b	.026
Total PUFAs	23.08 ± 4.49b	23.5 ± 2.82b	26.15 ± 4.28a	25.58 ± 4.35a	25.48 ± 4.10a	25.67 ± 4.08a	.001
*n*−6/*n*−3	8.58 ± 1.71d	9.70 ± 2.48c	10.23 ± 2.07bc	10.51 ± 2.73abc	10.75 ± 2.90ab	11.32 ± 1.90a	<.001
LA/ALA	15.74 ± 3.20	15.31 ± 4.45	14.65 ± 3.85	14.76 ± 4.55	14.56 ± 4.36	15.84 ± 3.72	.400
ARA/DHA	1.44 ± 0.37	1.49 ± 0.54	1.42 ± 0.47	1.53 ± 0.59	1.54 ± 0.47	1.55 ± 0.47	.644

Note

Results are presented as mean ± SD. Values within the same row not sharing a common letter are significantly different (*p* < .05).

Abbreviations: ALA, α‐linolenic acid; ARA, arachidonic acid; DHA, docosahexaeonoic acid; DPA, docosapentaenoic acid; LA, linoleic acid; MCSFA, medium‐chain saturated fatty acid; MUFA, monounsaturated fatty acid; OA, oleic acid; PA, palmitic acid; PUFA, polyunsaturated fatty acid; SFA, saturated fatty acid.

Most of the individual fatty acids in breast milk exhibited significant changes as lactation progressed. In the case of SFAs, the percentages of all individual MCSFAs, including C8:0, C10:0, C12:0, and C14:0, showed an overall rising tendency, while PA and C24:0 demonstrated a declining trend. C18:0, C20:0, and C22:0 were stable at all lactation durations. Among MUFAs, significantly higher OA, C20:1*n*‐9, C22:1*n*‐9, and C24:1*n*‐9, but lower C16:1*n*‐7 and C18:1t levels were observed in the breast milk of lactation 0‒5 days compared with those values in other periods. For PUFAs, the proportions of LA, ALA, and C18:3*n*‐6 increased remarkably from lactation 0‒5 days to 15‒30 days and then remained constant, while other fatty acids, including C20:2*n*‐6, C20:3*n*‐6, C20:4*n*‐6 (arachidonic acid, ARA), C22:2*n*‐6, C22:4*n*‐6, C20:5*n*‐3, C22:5*n*‐3 (docosapentaenoic acid, DPA), and C22:6*n*‐3 (docosahexaenoic acid, DHA), decreased significantly first and then maintained consistently. The ratio of *n*‐6/*n*‐3 led to a notable increment throughout the whole lactation period, from 8.58 in lactation 0‒5 days to 11.32 in lactation 181‒360 days. No significant variations were found in the ratios of LA/ALA (14.56‒15.84) and ARA/DHA (1.42‒1.55) among breast milk of different lactation intervals.

### 3.5 Sn‐2 fatty acid composition

Table 5 shows the *sn*‐2 fatty acid composition of human breast milk from different lactation periods. The amounts of total MCSFAs, SFAs, MUFAs, *n*‐6 PUFAs, *n*‐3 PUFAs, and PUFAs in the *sn*‐2 position of breast milk were 7.34%‒14.10%, 64.11%‒67.88%, 17.12%‒20.74%, 11.66%‒12.46%, 2.13%‒2.88%, and 14.15%‒14.84%, respectively. Significant variations were observed in most of the individual *sn*‐2 fatty acids (except C15:0, C18:0, C20:0, C22:0, C14:1*n*‐5, and C18:1t) among breast milk of different lactation periods, and the change trends were generally consistent with those of total fatty acids.

**TABLE 5:** *Sn*‐2 fatty acid composition (wt% of total *sn*‐2 fatty acids) of human breast milk from different lactation periods

Fatty acids	Lactation period						*p* value
	0–5 days	6–14 days	15–30 days	31–90 days	91–180 days	181–360 days	
C8:0	0.08 ± 0.07b	0.08 ± 0.06b	0.12 ± 0.08a	0.13 ± 0.10a	0.12 ± 0.07a	0.12 ± 0.08a	.012
C10:0	0.32 ± 0.20c	0.80 ± 0.29b	0.85 ± 0.37ab	0.93 ± 0.35ab	0.98 ± 0.41a	0.96 ± 0.38a	<.001
C12:0	2.24 ± 0.94e	4.13 ± 1.09 cd	3.76 ± 1.34d	4.55 ± 1.34bc	5.02 ± 1.37ab	5.46 ± 1.62a	<.001
C14:0	4.70 ± 1.59d	6.73 ± 1.96b	5.80 ± 1.66c	6.52 ± 1.78bc	6.95 ± 1.70ab	7.56 ± 2.03a	<.001
Total MCSFAs	7.34 ± 2.30e	11.73 ± 2.92c	10.53 ± 2.67d	12.12 ± 3.05bc	13.08 ± 2.91ab	14.10 ± 3.34a	<.001
C15:0	0.20 ± 0.13	0.21 ± 0.13	0.21 ± 0.12	0.19 ± 0.12	0.20 ± 0.11	0.22 ± 0.11	.758
C16:0 (PA)	50.88 ± 3.94a	49.80 ± 5.58ab	50.73 ± 4.90a	49.94 ± 4.12ab	48.54 ± 3.73b	48.21 ± 4.37b	.005
C17:0	0.38 ± 0.15c	0.41 ± 0.15bc	0.48 ± 0.16ab	0.49 ± 0.15a	0.47 ± 0.15ab	0.46 ± 0.20ab	.005
C18:0	4.29 ± 1.38	4.42 ± 1.50	4.29 ± 1.28	4.27 ± 1.24	4.32 ± 1.30	4.15 ± 1.23	.961
C20:0	0.20 ± 0.09	0.22 ± 0.09	0.21 ± 0.07	0.20 ± 0.07	0.20 ± 0.07	0.20 ± 0.06	.782
C22:0	0.33 ± 0.13	0.36 ± 0.09	0.32 ± 0.13	0.32 ± 0.13	0.36 ± 0.12	0.34 ± 0.11	.204
C24:0	0.49 ± 0.18a	0.37 ± 0.16b	0.26 ± 0.11c	0.26 ± 0.12c	0.24 ± 0.12 cd	0.20 ± 0.09d	<.001
Total SFAs	64.11 ± 4.05b	67.53 ± 4.51a	67.03 ± 4.24a	67.80 ± 4.09a	67.41 ± 3.84a	67.88 ± 3.67a	<.001
C14:1n−5	0.12 ± 0.11	0.14 ± 0.10	0.15 ± 0.13	0.15 ± 0.11	0.15 ± 0.10	0.14 ± 0.12	.758
C16:1n−7	1.58 ± 0.52b	1.70 ± 0.49b	1.99 ± 0.72a	1.96 ± 0.45a	1.93 ± 0.57a	1.92 ± 0.52a	.002
C18:1 t	0.11 ± 0.10	0.09 ± 0.07	0.12 ± 0.11	0.11 ± 0.11	0.09 ± 0.09	0.10 ± 0.08	.450
C18:1n−9 (OA)	17.59 ± 3.95a	15.06 ± 3.71b	14.72 ± 3.24b	14.53 ± 3.51b	14.59 ± 3.10b	14.37 ± 3.15b	<.001
C20:1n−9	0.51 ± 0.18a	0.34 ± 0.11b	0.33 ± 0.11b	0.31 ± 0.15b	0.29 ± 0.16b	0.28 ± 0.13b	<.001
C22:1n−9	0.32 ± 0.16a	0.21 ± 0.14b	0.20 ± 0.11b	0.17 ± 0.13bc	0.15 ± 0.11 cd	0.12 ± 0.06d	<.001
C24:1n−9	0.51 ± 0.17a	0.22 ± 0.10b	0.19 ± 0.11b	0.20 ± 0.09b	0.18 ± 0.09b	0.18 ± 0.06b	<.001
Total MUFAs	20.74 ± 4.12a	17.77 ± 3.89b	17.71 ± 3.29b	17.44 ± 3.51b	17.39 ± 3.12b	17.12 ± 3.25b	<.001
C18:2n−6 (LA)	8.35 ± 1.65c	9.33 ± 2.04b	10.63 ± 2.25a	10.20 ± 1.85a	10.76 ± 1.91a	10.64 ± 1.79a	<.001
C18:3n−6	0.13 ± 0.09b	0.12 ± 0.08b	0.19 ± 0.13a	0.21 ± 0.17a	0.19 ± 0.11a	0.20 ± 0.13a	.004
C20:2n−6	0.36 ± 0.10a	0.20 ± 0.10b	0.18 ± 0.07bc	0.17 ± 0.07bc	0.17 ± 0.07c	0.19 ± 0.09bc	<.001
C20:3n−6	0.35 ± 0.15a	0.24 ± 0.09b	0.20 ± 0.08bc	0.18 ± 0.12 cd	0.16 ± 0.10 cd	0.15 ± 0.10d	<.001
C20:4n−6 (ARA)	1.39 ± 0.30a	0.95 ± 0.20b	0.71 ± 0.26c	0.70 ± 0.20c	0.67 ± 0.20c	0.69 ± 0.22c	<.001
C22:2n−6	0.42 ± 0.18a	0.33 ± 0.14b	0.28 ± 0.11c	0.24 ± 0.11 cd	0.22 ± 0.08d	0.20 ± 0.08d	<.001
C22:4n−6	0.96 ± 0.38a	0.48 ± 0.16b	0.27 ± 0.12c	0.28 ± 0.13c	0.29 ± 0.11c	0.27 ± 0.14c	<.001
Total *n*−6 PUFAs	11.96 ± 1.83	11.66 ± 1.92	12.45 ± 2.23	11.99 ± 1.92	12.46 ± 1.92	12.34 ± 1.90	.300
C18:3n−3 (ALA)	0.64 ± 0.23c	0.82 ± 0.36b	1.00 ± 0.38a	1.02 ± 0.44a	1.07 ± 0.38a	0.99 ± 0.38a	<.001
C20:5n−3	0.11 ± 0.07a	0.08 ± 0.06ab	0.07 ± 0.06b	0.07 ± 0.05b	0.07 ± 0.05b	0.06 ± 0.07b	.002
C22:5n−3 (DPA)	0.81 ± 0.35a	0.49 ± 0.17b	0.41 ± 0.18c	0.39 ± 0.15c	0.38 ± 0.12c	0.37 ± 0.15c	<.001
C22:6n−3 (DHA)	1.33 ± 0.41a	1.10 ± 0.38b	0.83 ± 0.31c	0.77 ± 0.33 cd	0.69 ± 0.23d	0.71 ± 0.26 cd	<.001
Total *n*−3 PUFAs	2.88 ± 0.54a	2.49 ± 0.58b	2.31 ± 0.47bc	2.24 ± 0.60c	2.22 ± 0.41c	2.13 ± 0.57c	<.001
Total PUFAs	14.84 ± 2.15	14.15 ± 2.33	14.76 ± 2.48	14.23 ± 2.36	14.68 ± 2.13	14.47 ± 2.23	.605
*n*−6/*n*−3	4.23 ± 0.71d	4.82 ± 0.89c	5.54 ± 1.16b	5.59 ± 1.18b	5.76 ± 1.10ab	6.12 ± 1.58a	<.001
LA/ALA	13.88 ± 2.62a	13.36 ± 6.53ab	11.80 ± 3.51bc	11.44 ± 3.75c	11.02 ± 3.81c	12.02 ± 3.94bc	.004
ARA/DHA	1.12 ± 0.43	0.93 ± 0.47	0.91 ± 0.35	1.03 ± 0.41	1.05 ± 0.40	1.09 ± 0.41	.078

Note

Results are presented as mean ± SD. Values within the same row not sharing a common letter are significantly different (*p* < .05).

Abbreviations: ALA, α‐linolenic acid; ARA, arachidonic acid; DHA, docosahexaeonoic acid; DPA, docosapentaenoic acid; LA, linoleic acid; MCSFA, medium‐chain saturated fatty acid; MUFA, monounsaturated fatty acid; OA, oleic acid; PA, palmitic acid; PUFA, polyunsaturated fatty acid; SFA, saturated fatty acid.

### 3.6 Fatty acid positional distribution

**FIGURE 1:**
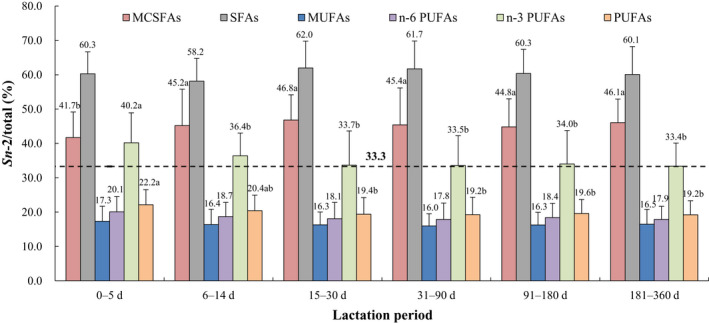
Positional distribution (*sn*‐2/total, %) of SFAs, MUFAs, and PUFAs in human breast milk from different lactation periods. Results are presented as mean ± SD. Values in the bars of the same type not sharing a common letter are significantly different (*p* < .05). MCSFA, medium‐chain saturated fatty acid; MUFA, monounsaturated fatty acids; PUFA, polyunsaturated fatty acid; SFA, saturated fatty acid

**FIGURE 2:**
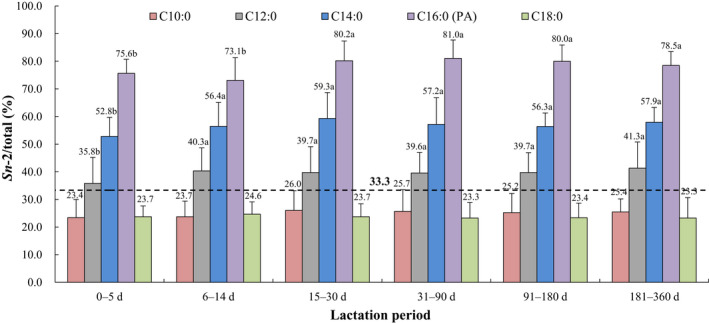
Positional distribution (*sn*‐2/total, %) of selected individual SFAs in human breast milk from different lactation periods. Results are presented as mean ± SD. Values in the bars of the same type not sharing a common letter are significantly different (*p* < .05). PA: palmitic acid

**FIGURE 3:**
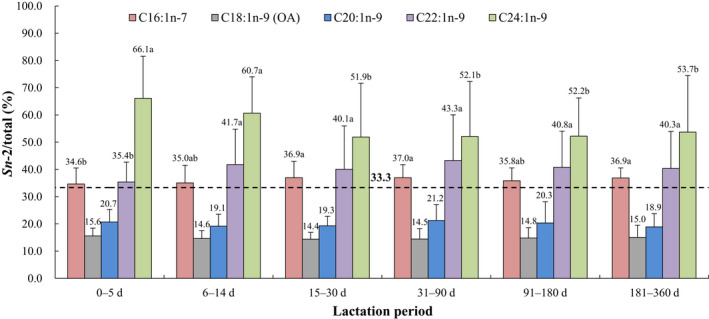
Positional distribution (*sn*‐2/total, %) of selected individual MUFAs in human breast milk from different lactation periods. Results are presented as mean ± SD. Values in the bars of the same type not sharing a common letter are significantly different (*p* < .05). OA, oleic acid

**FIGURE 4:**
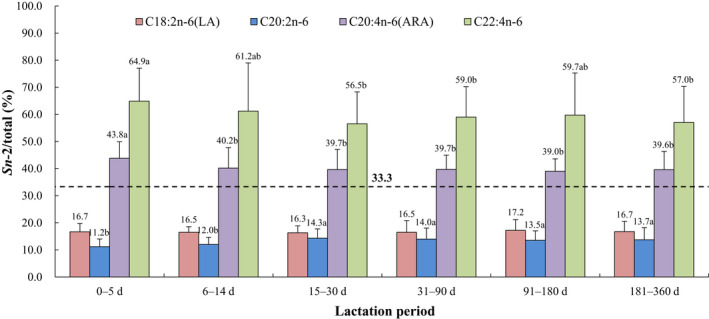
Positional distribution (*sn*‐2/total, %) of selected individual n‐6 PUFAs in human breast milk from different lactation periods. Results are presented as mean ± SD. Values in the bars of the same type not sharing a common letter are significantly different (*p* < .05). ARA, arachidonic acid; LA, linoleic acid

**FIGURE 5:**
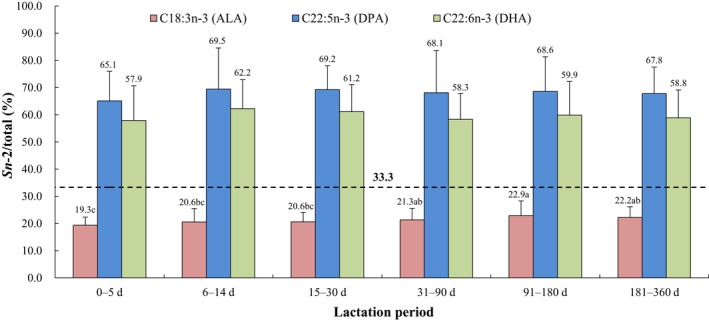
Positional distribution (*sn*‐2/total, %) of selected individual *n*‐3 PUFAs in human breast milk from different lactation periods. Results are presented as mean ± SD. Values in the bars of the same type not sharing a common letter are significantly different (*p* < .05). ALA, α‐linolenic acid; DHA, docosahexaeonoic acid; DPA, docosapentaenoic acid

The positional distributions of overall fatty acids and selected individual fatty acids (*sn*‐2/total, %, calculated as the relative percentage of a specific fatty acid at the *sn*‐2 position compared with the total amount of this fatty acid) in human breast milk from different lactation periods are presented in Figures 1, 2, 3, 4, 5. The relative percentages of total MCSFAs, SFAs, MUFAs, *n*‐6 PUFAs, *n*‐3 PUFAs, and PUFAs esterified in the *sn*‐2 position were 41.7%‒46.8%, 58.2%‒62.0%, 16.0%‒17.3%, 17.8%‒20.1%, 33.4%‒40.2%, and 19.2%‒22.2%, respectively. Milk samples of lactation 0‒5 days demonstrated significantly lower relative proportions of C12:0, C14:0, PA, C16:1*n*‐7, C22:1*n*‐9, C20:2*n*‐6, and ALA but higher relative proportions of C24:1*n*‐9, ARA, and C22:4*n*‐6 at the *sn*‐2 position than the milk samples of lactation over 31 days. The positional distributions of C10:0, C18:0, OA, C20:1*n*‐9, LA, DPA, and DHA were constant over the whole lactation. Regardless of the lactation period, C14:0, PA, C24:1*n*‐9, C22:4*n*‐6, DPA, and DHA were located mainly at the *sn*‐2 position, while C10:0, C18:0, OA, C20:1*n*‐9, LA, C20:2*n*‐6, and ALA were primarily distributed at the *sn*‐1,3 positions. No obvious positional preferences were found for C12:0, C16:1*n*‐7, C22:1*n*‐9, and ARA.

### 3.7 Nutritional composition after adjustment for basic characteristics of lactating mothers

In order to eliminate the influence of the difference of basic characteristics of lactating mothers on the nutritional composition of corresponding breast milk, the macronutrient content, lipid profile, fatty acid, and *sn*‐2 fatty acid composition of human breast milk from different lactation periods were adjusted for childbirth age, gestational period, pregestation, and predelivery BMI of sampled mothers (Table S1–S4). By comparison, the adjusted nutritional compositions and lactational changes of breast milk were consistent with those of unadjusted (Tables 1, 2, 3, 4).

## 4 DISCUSSION

### 4.1 Macronutrient content

Due to the importance of human milk composition in the estimation of infant nutritional requirements and the common use of a newly developed rapid analytic method (mid‐infrared spectrometry) for the determination of human milk macronutrients (Fusch et al., 2014), the lipid, protein, and lactose content of human milk from different regions and lactation periods have been widely investigated in recent years (Gidrewicz & Fenton, 2014; Leghi et al., 2020). Among macronutrients, the lipid content was directly affected by the sampling mode of human milk. Foremilk was reported to have a much higher lipid content (>2‐fold) than hindmilk (Mitoulas et al., 2002). Therefore, whole milk samples should be used when studying the lipid content of breast milk. In the present study, whole milk samples were collected and the lipid, protein, and lactose concentrations were 2.81‒4.05, 1.07‒2.19, and 6.63‒6.97 g/100 ml, respectively. This macronutrient composition was different from the macronutrient composition of whole human milk in many Western countries, including the USA (2.67‒3.19, 0.96‒1.32, and 5.90‒6.39 g/100 ml), Italy (2.71‒3.20, 0.96‒1.38, and 7.24‒8.03 g/100 ml), Australia (3.52‒4.09, 0.80‒1.05, and 5.97‒6.28 g/100 ml), and Switzerland (2.1‒3.7, 1.7‒2.5, and 5.6‒6.1 g/100 ml; Fischer Fumeaux et al., 2019; Grote et al., 2015; Mitoulas et al., 2002; Sauer et al., 2017), but comparable to the macronutrient composition in Asian countries, such as Japan and Korea (2.68‒3.90, 0.99‒2.20, 5.59‒7.10 g/100 ml; Chang et al., 2015; Yamawaki et al., 2005). Among the macronutrients, lipid and protein levels were different depending on the periods of lactation with a large interindividual variation. For lipids, the concentration was higher in the later periods of lactation (>15 days) compared to the lipid concentration at 0‒5 and 6‒14 days, whereas the protein level constantly decreased from 0 to 5 days until 181‒360 days with statistical significance. This change tendency is in line with the previous studies that also showed a large difference in lipid and protein contents during lactation (Gidrewicz & Fenton, 2014; Leghi et al., 2020).

### 4.2 Lipid profile

Human breast milk lipids are complex and exist mainly in the form of milk fat globules, which contain a core of nonpolar lipids comprised primarily of TAGs, with small amounts of MAGs, DAGs, and FFAs, and a trilayer membrane of polar lipids comprised primarily of PLs and CHOLs including FCHOLs and CEs (Koletzko, 2016). In the present study, TAGs were the most abundant lipid class in all milk samples, accounting for over 97% of the total lipids. PLs was the second‐largest lipid fraction, with an average percentage of 1%, while CEs, FCHOLs, MAGs, DAGs, and FFAs were all in small proportions (less than 0.50%). This pattern of lipid composition was in line with those reported in other studies (Koletzko, 2016; Liu et al., 2018).

From our data, all lipid classes underwent significant lactational changes in human milk within the first month after childbirth. As lactation was prolonged from 0‒5 to 15‒30 days, the percentage of TAGs in total lipids showed a significant but small increment (from 97.15% to 97.72%), whereas PLs, CEs, and FCHOLs decreased by over 40% (from 1.32%, 0.49%, 0.32% to 0.73%, 0.27%, 0.19%, respectively). Similar large decreases in the contents of PLs and CHOLs were observed in the previous reports. In the case of PL content, human milk from Shanghai (China) (Wu et al., 2019) and Granada (Spain) (Sala‐Vila et al., 2005) demonstrated a decrease of approximately 20%‒25% with the prolongation of lactation from 0‒5 days to >15 days (41‒45 and 16‒30 days). For CHOL concentration, Hamdan et al. (Hamdan et al., 2018) found a decline of over 40% in human milk as lactation progressed from 0‒5 to 16‒60 days. These descending trends can be partially explained by the idea that in the later stages of lactation, the biosynthesis of PLs and CHOLs in the mammary gland diminishes (Hamdan et al., 2018). In addition, the decreasing size of milk fat globules with prolonged lactation may also contribute to the decline of PL percentages in human milk (Zou et al., 2012).

### 4.3 Fatty acid composition

The fatty acid composition of human milk was evidenced to vary with region and was susceptible to maternal diet (Jiang et al., 2016; Yuhas et al., 2006). Milk samples of the mature stage (lactation days >15 days) in our study which showed similar fatty acid profile with those in neighboring cities (Shanghai, Wuxi) of China (Qi et al., 2018; Wu et al., 2019), had higher percentages of PUFAs (25.48%‒26.15%), LA (21.40%‒21.87%), ALA (1.44%‒1.59%), ARA (0.58%‒0.63%), and DHA (0.40%‒0.48%), but lower levels of MCSFAs (7.81%‒10.45%), SFAs (36.45%‒38.16%), C12:0 (3.27%‒4.61%), and C14:0 (3.39%‒4.41%) than those in some Western countries including Canada, Australia, England, and German, which contained a range of 13.68%‒17.19%, 10.45%‒13.62%, 0.87%‒1.22%, 0.35%‒0.43%, 0.17%‒0.36%, 12.95%‒13.63%, 38.47%‒44.66%, 4.80%‒5.90%, and 5.84%‒7.87% for the fatty acids, respectively (Gardner et al., 2017; Miliku et al., 2019; Much et al., 2013; Yuhas et al., 2006). Mediterranean countries such as Italy and Spain were reported to have much higher milk MUFAs (38.59%‒47.00%) contents than our study (35.58%‒36.82%) (Grote et al., 2015; López‐López et al., 2002), while some coastal island countries such as Japan and the Philippines demonstrated a relatively high proportion of DHA (0.74%‒0.99%) in human milk compared with our data (0.40%‒0.48%; Yuhas et al., 2006). These variations are attributed mainly to the different regional dietary habits. In Western countries, people tend to consume more animal food, which contains high amounts of SFAs but low levels of PUFAs, in their daily life (Jiang et al., 2016; Wu et al., 2019). However, the Chinese daily diet is characterized by high intakes of vegetable oils, especially soybean oil and corn oil, which are rich in LA and ALA, the precursors of *n*‐6 and *n*‐3 PUFAs (Jiang et al., 2016). The Mediterranean diet contains large quantities of olive oil, which is high in OA (López‐López et al., 2002), while coastal residents eat more seafood rich in DHA (Fu et al., 2016; Yuhas et al., 2006). Compared with the human milk in Western countries (the USA, Canada, and Germany), samples in the present study demonstrated higher *n*‐6/*n*‐3 and LA/ALA, but lower AA/DHA ratios (8.58‒11.32 vs. 6.53‒8.36, 14.56‒15.84 vs. 7.09‒12.31 and 1.42‒1.55 vs. 1.77‒3.43; Martin et al., 2012; Miliku et al., 2019; Much et al., 2013).

Apart from regional variations, lactational changes in the fatty acid composition of human milk have also been widely reported (Floris et al., 2020; Koletzko, 2016). From our study, most fatty acids in human milk underwent significant differences as the lactation period progressed from 0‒5 to 15‒30 days. Milk samples of lactation 0‒5 days demonstrated significantly higher levels of PA, C24:0, OA, C24:1*n*‐9, C20:2*n*‐6, C20:3*n*‐6, C22:2*n*‐6, ARA, DPA, and DHA but lower contents of C10:0, C12:0, C14:0, LA, and ALA compared to those in 15‒30 days. This change in pattern, which agreed with most of the other previous studies (Floris et al., 2020), is related mainly to the physiological characteristics and nutrition needs of lactating mothers and infants in different periods (Jiang et al., 2016). With the further extension of the lactation period, no significant variation was observed in most of the corresponding fatty acids except C12:0 and C14:0, which led to continuous increments from 3.91% and 3.78% in lactation 31‒60 days to 4.61% and 4.41% in lactation 181‒360 days. Hence, the significant variations of MCSFA content in human milk among different periods of one‐year lactation should be taken into consideration when developing infant formulas of different stages.

### 4.4 Fatty acid positional distribution

In addition to the fatty acid composition, the specified positional distribution of fatty acids on the triglyceride structure of human milk is another key point (Wei et al., 2019). From the present study, up to 75.6%‒81.0% of PA (the most abundant SFA) was located at the *sn*‐2 position, whereas the relative percentage of OA (the predominant MUFA) esterified at the *sn*‐2 position was only 14.4%‒15.6%. By comparison, a previous Chinese study conducted in Shanghai city demonstrated similar proportions of PA (74.65%‒80.75%) and slightly lower OA (11.70%‒13.62%) (Wu et al., 2019), while another study carried out in Inner Mongolia, North Jiangsu and Guangxi districts showed higher PA (86.33%‒93.82%) and slightly lower OA (11.12%‒13.28%; Deng et al., 2018). In contrast to foreign studies, the relative percentage of PA at the *sn*‐2 position was higher in Spain (80.30%‒87.86%) and lower in Italy (59.11%‒62.00%), but the levels of OA (Spain: 12.22%‒14.10%, Italy: 9.83%‒15.80%) were comparable to our data (Haddad et al., 2012; López‐López et al., 2002).

Although the relative percentages of PA and OA at the *sn*‐2 position of human milk varied with region, it was certain that PA existed primarily at the *sn*‐2 position, and OA esterified mainly at the *sn*‐1,3 positions. These typical positional preferences make OA‐PA‐OA (OPO) one of the most abundant TAGs in human milk (Haddad et al., 2012; Morera Pons et al., 2000) and have been widely proven to be positively related to better fatty acid and mineral absorption and less hard stool and constipation incidence in infants (Bar‐Yoseph et al., 2013; López‐López et al., 2001).

In terms of PUFAs, our study showed that both LA and ALA were preferentially distributed at the *sn*‐1,3 positions (16.3%‒17.2% and 14.4%‒15.6% at the *sn*‐2 position), while DPA, DHA, and C22:4*n*‐6 were selectively located at the *sn*‐2 position (65.1%‒69.5%, 57.9%‒62.2%, and 57.0%‒64.9%), and ARA had no obvious positional preference (39.0%‒43.8%). Similar positional distribution patterns with different relative proportions were observed in several previous studies. A recent Chinese study conducted in Shanghai, as well as a foreign study carried out in Spain, demonstrated higher relative percentages of LA (20.03%‒23.84%) and ALA (23.90%‒26.67%) and comparable levels of DPA (62.16%‒72.29%), DHA (52.63%‒61.89%), C22:4n‐6 (61.32%‒73.48%), and ARA (30.84%‒47.85%) at the *sn*‐2 position (López‐López et al., 2002; Wu et al., 2019). Due to the higher levels of LA and its *sn*‐1,3 positional preferences, Chinese human milk has been reported to contain more OA–PA–LA (OPL) than OPO (Tu et al., 2017; Zhang et al., 2019), which is the most abundant TAG in Western human milk. Therefore, the OPL structure needs to be considered more than the OPO structure when developing commercial infant formulas suitable for Chinese babies. The *sn*‐2 positional selectivity for DPA, DHA, and C22:4*n*‐6 in human milk has been well evidenced to be positively correlated with improved oxidative stability (Wijesundera, 2008) and improved absorption and enrichment in brain tissues (Christensen et al., 1995, 1998). Recently, technologies for producing TAG with *sn*‐2 DHA have garnered increasing attention (Álvarez & Akoh, 2016; Moreno‐Perez et al., 2017). These technologies may be applicable to the commercial production of TAGs that mimic those in breast milk for infant formulas.

According to the present study and some previous studies (López‐López et al., 2002; Wu et al., 2019), the carbon chain length and degree of unsaturation appeared to be discriminating factors in the positional distribution of the fatty acids. The relative proportions of SFAs and MUFAs at the *sn*‐2 position increased considerably from C10:0 to C16:0 and C18:1 to C24:1, respectively. The percentages of relative PUFAs in the *sn*‐2 position showed a rising trend from C18:2 to C22:6.

Although human milk is generally considered to be the most suitable food with perfectly balanced nutrition for the growing infant, its nutritional composition varies greatly among different individuals, regions, and lactation periods. Therefore, when studying the nutritional composition of human milk, the size of milk samples, the selection of collection regions, and the coverage of lactation periods should be as representative as possible. In this study, the sample size of human milk reached 378 cases; the collection region was selected from one of the most developed provinces; and the lactation periods covered a long range of 0‒360 days. Compared with the previous studies, the present study provided more useful data for the development of infant formula closer to Chinese breast milk in terms of macronutrient content, lipid profile, fatty acid composition, and positional distribution. However, this study is limited to one of the numerous developed regions in China and does not completely represent the levels of whole Chinese breast milk. Further work is needed to properly characterize other regions.

## 5 CONCLUSIONS

The present study investigated the macronutrient content, lipid profile, fatty acid composition, and positional distribution in the human breast milk of six different lactation periods (0‒5, 6‒14, 15‒30, 31‒90, 91‒180, and 181‒360 days) collected cross‐sectionally from Zhejiang, China. For breast milk within the first month postpartum (0‒5, 6‒14, and 15‒30 days), most of the analyzed nutrients underwent significant variations with the extension of the lactation period. Milk samples in lactation 0‒5 days demonstrated the significantly higher concentration of protein and proportions of PLs, CHOLs, PA, OA, C24:1*n*‐9, ARA, C22:4*n*‐6, DPA, and DHA, but lower content of lipids and percentages of C10:0, C12:0, C14:0, LA, and ALA than those in lactation 15‒30 days. However, for breast milk with a lactation period over one month (31‒90, 91‒180, and 181‒360 days), no significant change was observed in most of the nutrition compositions except protein content and C12:0, C14:0 percentages, which led a continuous decreasing and increasing trend, respectively. Compared with the fatty acid composition, the fatty acid positional distribution was much less varied among human milk samples of different lactation periods. Regardless of lactational variations, C14:0, PA, C24:1*n*‐9, C22:4*n*‐6, DPA, and DHA were located mainly at the *sn*‐2 position, while OA, LA, and ALA were primarily distributed at the *sn*‐1,3 positions.

In China, the studies of human milk composition are not systematic and comprehensive enough. Meanwhile, the research and industrialization of human milk‐simulated infant formula are still in the primary stage, especially for the simulation of the proportion of fatty acids in the *sn*‐2 position of triglyceride. The present study provides significant insights into the standard for the lipid configuration of infant formula and will aid the development of localized infant formulas with optimal lipid composition and structure close to the Chinese human milk.

## CONFLICT OF INTERESTS

The authors declare that they do not have any conflict of interest.

## AUTHOR CONTRIBUTIONS

**Guanghua He:** Conceptualization (supporting); Data curation (supporting); Formal analysis (lead); Funding acquisition (supporting); Investigation (lead); Methodology (lead); Writing‐original draft (supporting). **Guipu Li:** Data curation (supporting); Formal analysis (supporting); Investigation (supporting); Methodology (supporting); Visualization (lead); Writing‐original draft (lead). **Yanxi Jiang:** Data curation (supporting); Investigation (supporting); Resources (lead). **Jiacai Hua:** Data curation (supporting); Formal analysis (supporting); Resources (supporting). **Xiaojun Chu:** Conceptualization (supporting); Methodology (supporting); Visualization (supporting). **Lina Xiong:** Data curation (supporting); Project administration (supporting); Writing‐original draft (supporting). **Jinyan Gong:** Conceptualization (supporting); Data curation (supporting); Formal analysis (supporting). **Gongnian Xiao:** Data curation (supporting); Investigation (supporting); Methodology (supporting). **Xingqian Ye:** Conceptualization (lead); Funding acquisition (lead); Project administration (lead); Supervision (lead); Visualization (lead); Writing‐review & editing (lead).

## ETHICAL STATEMENT

The study protocol was approved by the Ethics Committee of the College of Biosystem Engineering and Food Science, Zhejiang University. Written informed consent was obtained from all study participants. The study conforms to the principles outlined in the Declaration of Helsinki.

## Supporting information

Table S1‐S4
