# Supplementary material for: Macronutrient content and fatty acid composition and their positional distribution in human breast milk from Zhejiang Province, China in different lactation periods
Source: Food Sci Nutr. 2021 Oct 20;9(12):6746–61. doi: 10.1002/fsn3.2626 (PMC8645764; doi:10.1002/fsn3.2626)
Supplement: Supplementary file 1 — Table S1‐S4 [file FSN3-9-6746-s001.docx]

**Table S1.** Macronutrient contents (g/100 mL) of human breast milk from different lactation periods after adjustment for childbirth age, gestational period, pregestation and predelivery BMI of lactating mothers

| Macronutrients | Lactation period | | | | | | | | | | | | *P* value |
| --- | --- | --- | --- | --- | --- | --- | --- | --- | --- | --- | --- | --- | --- |
|  | 0‒5 d | | 6‒14 d | | 15‒30 d | | 31‒90 d | | 91‒180 d | | 181‒360 d | |  |
|  | mean | 95% CI | mean | 95% CI | mean | 95% CI | mean | 95% CI | mean | 95% CI | mean | 95% CI |  |
| Lipid | 2.83b | 2.50‒3.16 | 3.51ab | 3.16‒3.85 | 3.80a | 3.52‒4.08 | 4.03a | 3.78‒4.29 | 3.88a | 3.63‒4.14 | 4.02a | 3.75‒4.29 | <0.001 |
| Protein | 2.19a | 2.10‒2.27 | 1.77b | 1.67‒1.86 | 1.45c | 1.37‒1.52 | 1.22d | 1.15‒1.29 | 1.14de | 1.07‒1.21 | 1.07e | 1.01‒1.14 | <0.001 |
| Lactose | 6.64 | 6.43‒6.85 | 6.86 | 6.75‒6.98 | 6.95 | 6.86‒7.04 | 6.90 | 6.81‒6.99 | 6.98 | 6.89‒7.07 | 6.88 | 6.79‒6.97 | 0.127 |

Results are presented as mean and 95% confidence interval (CI). Mean values within the same row not sharing a common letter are significantly different (*P*<0.05).

**Table S2.** Lipid profile (wt% of total lipids) of human breast milk from different lactation periods after adjustment for childbirth age, gestational period, pregestation and predelivery BMI of lactating mothers

| Lipid class | Lactation period | | | | | | | | | | | | *P* value |
| --- | --- | --- | --- | --- | --- | --- | --- | --- | --- | --- | --- | --- | --- |
|  | 0‒5 d | | 6‒14 d | | 15‒30 d | | 31‒90 d | | 91‒180 d | | 181‒360 d | |  |
|  | mean | 95% CI | mean | 95% CI | mean | 95% CI | mean | 95% CI | mean | 95% CI | mean | 95% CI |  |
| TAGs | 97.15c | 97.06‒97.24 | 97.47b | 97.38‒97.57 | 97.73a | 97.65‒97.81 | 97.78a | 97.70‒97.84 | 97.66a | 97.59‒97.73 | 97.73a | 97.66‒97.81 | <0.001 |
| PLs | 1.32a | 1.27‒1.37 | 0.90b | 0.85‒0.96 | 0.73c | 0.68‒0.77 | 0.71c | 0.67‒0.74 | 0.74c | 0.70‒0.78 | 0.68c | 0.64‒0.72 | <0.001 |
| CEs | 0.49a | 0.46‒0.52 | 0.31b | 0.27‒0.34 | 0.27b | 0.24‒0.30 | 0.28b | 0.26‒0.31 | 0.28b | 0.26‒0.31 | 0.29b | 0.26‒0.32 | <0.001 |
| FCHOLs | 0.32a | 0.29‒0.34 | 0.25b | 0.22‒0.28 | 0.19c | 0.16‒0.21 | 0.19c | 0.17‒0.21 | 0.21bc | 0.19‒0.23 | 0.20bc | 0.17‒0.22 | <0.001 |
| MAGs | 0.21b | 0.18‒0.23 | 0.25ab | 0.22‒0.28 | 0.28a | 0.26‒0.30 | 0.28a | 0.26‒0.30 | 0.30a | 0.28‒0.32 | 0.28a | 0.25‒0.30 | <0.001 |
| 1, 2-DAGs | 0.13b | 0.11‒0.15 | 0.22a | 0.20‒0.24 | 0.22a | 0.20‒0.23 | 0.21a | 0.19‒0.22 | 0.21a | 0.20‒0.23 | 0.23a | 0.21‒0.24 | <0.001 |
| 1, 3-DAGs | 0.15c | 0.13‒0.17 | 0.21ab | 0.19‒0.23 | 0.22a | 0.20‒0.24 | 0.18bc | 0.16‒0.19 | 0.20ab | 0.18‒0.21 | 0.22a | 0.20‒0.24 | <0.001 |
| FFAs | 0.24b | 0.22‒0.27 | 0.39a | 0.36‒0.42 | 0.38a | 0.35‒0.40 | 0.38a | 0.36‒0.40 | 0.40a | 0.38‒0.42 | 0.37a | 0.35‒0.40 | <0.001 |

Results are presented as mean and 95% confidence interval (CI). Mean values within the same row not sharing a common letter are significantly different (*P*<0.05). TAG: triacylglycerols; PL: phospholipid; CE: cholesterol ester; FCHOL: free cholesterol; MAG: monoacylglycerol; DAGs: diacylglycerol; FFA: free fatty acid.

**Table S3.** Fatty acid composition (wt% of total fatty acids) of human breast milk from different lactation periods after adjustment for childbirth age, gestational period, pregestation and predelivery BMI of lactating mothers

| Fatty acids | Lactation period | | | | | | | | | | | | *P* value |
| --- | --- | --- | --- | --- | --- | --- | --- | --- | --- | --- | --- | --- | --- |
|  | 0‒5 d | | 6‒14 d | | 15‒30 d | | 31‒90 d | | 91‒180 d | | 181‒360 d | |  |
|  | mean | 95% CI | mean | 95% CI | mean | 95% CI | mean | 95% CI | mean | 95% CI | mean | 95% CI |  |
| C8:0 | 0.13b | 0.09‒0.16 | 0.14ab | 0.10‒0.18 | 0.21a | 0.18‒0.24 | 0.19ab | 0.16‒0.22 | 0.19ab | 0.16‒0.22 | 0.19ab | 0.16‒0.22 | 0.003 |
| C10:0 | 0.50b | 0.37‒0.62 | 1.20a | 1.06‒1.34 | 1.14a | 1.02‒1.25 | 1.26a | 1.15‒1.36 | 1.28a | 1.17‒1.38 | 1.24a | 1.12‒1.35 | <0.001 |
| C12:0 | 2.35d | 1.96‒2.73 | 3.67bc | 3.23‒4.10 | 3.30c | 2.93‒3.66 | 3.94abc | 3.61‒4.27 | 4.28ab | 3.95‒4.61 | 4.60a | 4.25‒4.95 | <0.001 |
| C14:0 | 3.02d | 2.67‒3.37 | 4.03abc | 3.64‒4.42 | 3.41cd | 3.08‒3.73 | 3.79abc | 3.50‒4.09 | 4.16ab | 3.86‒4.45 | 4.39a | 4.08‒4.70 | <0.001 |
| Total MCSFAs | 5.99c | 5.22‒6.76 | 9.04ab | 8.17‒9.90 | 7.86b | 7.14‒8.58 | 9.19ab | 8.54‒9.83 | 9.91a | 9.25‒10.56 | 10.41a | 9.72‒11.10 | <0.001 |
| C15:0 | 0.12 | 0.11‒0.14 | 0.11 | 0.10‒0.13 | 0.10 | 0.09‒0.12 | 0.10 | 0.08‒0.11 | 0.10 | 0.09‒0.12 | 0.11 | 0.09‒0.12 | 0.070 |
| C16:0 (PA) | 22.54a | 21.83‒23.25 | 22.76a | 21.97‒23.56 | 21.31ab | 20.64‒21.97 | 20.61b | 20.01‒21.21 | 20.39b | 19.79‒21.00 | 20.56b | 19.92‒21.20 | <0.001 |
| C17:0 | 0.27b | 0.25‒0.29 | 0.28ab | 0.26‒0.30 | 0.31a | 0.29‒0.33 | 0.31a | 0.29‒0.33 | 0.28ab | 0.26‒0.29 | 0.28ab | 0.26‒0.30 | 0.002 |
| C18:0 | 5.93 | 5.56‒6.30 | 6.03 | 5.62‒6.44 | 5.94 | 5.60‒6.28 | 6.10 | 5.79‒6.41 | 6.17 | 5.86‒6.48 | 6.07 | 5.74‒6.39 | 0.910 |
| C20:0 | 0.25 | 0.23‒0.27 | 0.27 | 0.24‒0.29 | 0.26 | 0.24‒0.28 | 0.27 | 0.25‒0.29 | 0.28 | 0.26‒0.30 | 0.27 | 0.25‒0.29 | 0.517 |
| C22:0 | 0.35 | 0.30‒0.40 | 0.39 | 0.33‒0.44 | 0.34 | 0.29‒0.38 | 0.32 | 0.28‒0.36 | 0.37 | 0.33‒0.41 | 0.35 | 0.30‒0.39 | 0.417 |
| C24:0 | 0.33a | 0.30‒0.36 | 0.21b | 0.18‒0.24 | 0.16bc | 0.13‒0.19 | 0.13c | 0.10‒0.15 | 0.14c | 0.12‒0.17 | 0.12c | 0.10‒0.15 | <0.001 |
| Total SFAs | 35.79c | 34.61‒36.96 | 39.09a | 37.77‒40.40 | 36.47bc | 35.37‒37.56 | 37.02abc | 36.03‒38.01 | 37.64abc | 36.64‒38.64 | 38.17ab | 37.11‒39.22 | 0.002 |
| C14:1n-5 | 0.15 | 0.12‒0.18 | 0.14 | 0.11‒0.17 | 0.11 | 0.08‒0.13 | 0.15 | 0.12‒0.17 | 0.13 | 0.10‒0.15 | 0.11 | 0.09‒0.14 | 0.085 |
| C16:1n-7 | 1.49b | 1.35‒1.63 | 1.64ab | 1.48‒1.80 | 1.82a | 1.68‒1.95 | 1.82a | 1.70‒1.94 | 1.76a | 1.64‒1.88 | 1.77a | 1.64‒1.89 | 0.005 |
| C18:1t | 0.10b | 0.08‒0.13 | 0.11ab | 0.08‒0.14 | 0.13ab | 0.11‒0.16 | 0.12ab | 0.10‒0.14 | 0.13ab | 0.11‒0.15 | 0.15a | 0.13‒0.17 | 0.029 |
| C18:1n-9 (OA) | 37.55a | 36.22‒38.87 | 34.03b | 32.55‒35.52 | 33.90b | 32.66‒35.13 | 33.89b | 32.77‒35.01 | 33.47b | 32.34‒34.60 | 32.85b | 31.66‒34.05 | <0.001 |
| C20:1n-9 | 0.85a | 0.80‒0.91 | 0.59b | 0.53‒0.65 | 0.56bc | 0.51‒0.61 | 0.48c | 0.43‒0.52 | 0.49bc | 0.44‒0.53 | 0.47c | 0.42‒0.51 | <0.001 |
| C22:1n-9 | 0.29a | 0.26‒0.32 | 0.18bc | 0.14‒0.21 | 0.17bc | 0.14‒0.19 | 0.13cd | 0.10‒0.15 | 0.12cd | 0.10‒0.15 | 0.10d | 0.08‒0.13 | <0.001 |
| C24:1n-9 | 0.26a | 0.24‒0.28 | 0.13b | 0.10‒0.15 | 0.13b | 0.11‒0.15 | 0.13b | 0.11‒0.15 | 0.13b | 0.11‒0.14 | 0.11b | 0.09‒0.13 | <0.001 |
| Total MUFAs | 40.69a | 39.34‒42.04 | 36.81b | 35.30‒38.33 | 36.81b | 35.55‒38.08 | 36.71b | 35.57‒37.85 | 36.23b | 35.08‒37.38 | 35.56b | 34.35‒36.78 | <0.001 |
| C18:2n-6 (LA) | 17.06b | 15.89‒18.23 | 18.82b | 17.51‒20.13 | 21.86a | 20.76‒22.95 | 21.48a | 20.50‒22.47 | 21.36a | 20.37‒22.36 | 21.75a | 20.70‒22.80 | <0.001 |
| C18:3n-6 | 0.19b | 0.16‒0.21 | 0.16b | 0.13‒0.19 | 0.29a | 0.26‒0.31 | 0.29a | 0.27‒0.32 | 0.30a | 0.28‒0.33 | 0.30a | 0.27‒0.33 | <0.001 |
| C20:2n-6 | 1.11a | 1.06‒1.15 | 0.55b | 0.49‒0.61 | 0.43c | 0.39‒0.48 | 0.41c | 0.37‒0.45 | 0.40c | 0.36‒0.44 | 0.44c | 0.41‒0.48 | <0.001 |
| C20:3n-6 | 0.46a | 0.43‒0.49 | 0.35b | 0.31‒0.38 | 0.28c | 0.25‒0.31 | 0.25cd | 0.23‒0.28 | 0.23cd | 0.21‒0.25 | 0.21d | 0.18‒0.23 | <0.001 |
| C20:4n-6 (ARA) | 1.08a | 1.01‒1.15 | 0.84b | 0.76‒0.92 | 0.63c | 0.56‒0.70 | 0.62c | 0.56‒0.68 | 0.57c | 0.51‒0.63 | 0.60c | 0.53‒0.67 | <0.001 |
| C22:2n-6 | 0.23a | 0.21‒0.24 | 0.17b | 0.15‒0.18 | 0.13c | 0.12‒0.15 | 0.11cd | 0.10‒0.13 | 0.10d | 0.08‒0.11 | 0.09d | 0.08‒0.11 | <0.001 |
| C22:4n-6 | 0.50a | 0.46‒0.53 | 0.28b | 0.24‒0.32 | 0.16c | 0.13‒0.19 | 0.17c | 0.14‒0.20 | 0.18c | 0.15‒0.21 | 0.16c | 0.12‒0.19 | <0.001 |
| Total n-6 PUFAs | 20.61c | 19.44‒21.78 | 21.17bc | 19.86‒22.47 | 23.78a | 22.69‒24.86 | 23.35ab | 22.37‒24.33 | 23.15ab | 22.15‒24.14 | 23.55a | 22.50‒24.59 | <0.001 |
| C18:3n-3 (ALA) | 1.11c | 0.99‒1.23 | 1.31bc | 1.18‒1.44 | 1.56a | 1.45‒1.67 | 1.55a | 1.45‒1.64 | 1.59a | 1.49‒1.69 | 1.44ab | 1.34‒1.55 | <0.001 |
| C20:5n-3 | 0.14a | 0.12‒0.16 | 0.12ab | 0.10‒0.15 | 0.11ab | 0.09‒0.13 | 0.11ab | 0.09‒0.13 | 0.10ab | 0.09‒0.12 | 0.09b | 0.07‒0.11 | 0.044 |
| C22:5n-3 (DPA) | 0.42a | 0.39‒0.46 | 0.25ab | 0.21‒0.28 | 0.21ab | 0.17‒0.24 | 0.20ab | 0.17‒0.23 | 0.20ab | 0.17‒0.22 | 0.19b | 0.16‒0.22 | <0.001 |
| C22:6n-3 (DHA) | 0.79a | 0.73‒0.86 | 0.61b | 0.54‒0.69 | 0.48bc | 0.42‒0.54 | 0.45c | 0.40‒0.50 | 0.40c | 0.35‒0.46 | 0.41c | 0.35‒0.46 | <0.001 |
| Total n-3 PUFAs | 2.47a | 2.32‒2.61 | 2.30ab | 2.13‒2.46 | 2.35ab | 2.22‒2.49 | 2.30ab | 2.18‒2.43 | 2.29ab | 2.17‒2.41 | 2.13b | 2.00‒2.26 | 0.029 |
| Total PUFAs | 23.08c | 21.86‒24.29 | 23.46bc | 22.11‒24.82 | 26.13a | 25.00‒27.26 | 25.65ab | 24.63‒26.67 | 25.44ab | 24.40‒26.47 | 25.67ab | 24.58‒26.76 | 0.001 |
| n-6/n-3 | 8.55c | 7.83‒9.27 | 9.72bc | 8.92‒10.53 | 10.27ab | 9.60‒10.94 | 10.52ab | 9.91‒11.12 | 10.73ab | 10.11‒11.34 | 11.31a | 10.67‒11.96 | <0.001 |
| LA/ALA | 15.65 | 14.45‒16.86 | 15.37 | 14.02‒16.72 | 14.75 | 13.62‒15.87 | 14.82 | 13.81‒15.83 | 14.52 | 13.49‒15.54 | 15.77 | 14.69‒16.85 | 0.491 |
| ARA/DHA | 1.44 | 1.29‒1.58 | 1.49 | 1.33‒1.65 | 1.42 | 1.29‒1.56 | 1.54 | 1.41‒1.66 | 1.52 | 1.40‒1.65 | 1.56 | 1.43‒1.69 | 0.636 |

Results are presented as mean and 95% confidence interval (CI). Mean values within the same row not sharing a common letter are significantly different (*P*<0.05). MCSFA: medium-chain saturated fatty acid; SFA: saturated fatty acid; MUFA: monounsaturated fatty acid; PUFA: polyunsaturated fatty acid; PA: palmitic acid; OA: oleic acid; LA: linoleic acid; ARA: arachidonic acid; ALA: α-linolenic acid; DPA: docosapentaenoic acid; DHA: docosahexaeonoic acid.

**Table S4.** *Sn*-2 fatty acid composition (wt% of total *sn*-2 fatty acids) of human breast milk from different lactation periods after adjustment for childbirth age, gestational period, pregestation and predelivery BMI of lactating mothers

| Fatty acids | Lactation period | | | | | | | | | | | | *P* value |
| --- | --- | --- | --- | --- | --- | --- | --- | --- | --- | --- | --- | --- | --- |
|  | 0‒5 d | | 6‒14 d | | 15‒30 d | | 31‒90 d | | 91‒180 d | | 181‒360 d | |  |
|  | mean | 95% CI | mean | 95% CI | mean | 95% CI | mean | 95% CI | mean | 95% CI | mean | 95% CI |  |
| C8:0 | 0.08b | 0.06‒0.11 | 0.08ab | 0.05‒0.11 | 0.12ab | 0.10‒0.14 | 0.13a | 0.11‒0.15 | 0.12ab | 0.10‒0.14 | 0.13ab | 0.11‒0.15 | 0.009 |
| C10:0 | 0.32b | 0.21‒0.44 | 0.79a | 0.66‒0.92 | 0.85a | 0.74‒0.95 | 0.93a | 0.84‒1.01 | 0.98a | 0.90‒1.07 | 0.96a | 0.87‒1.05 | <0.001 |
| C12:0 | 2.23e | 1.80‒2.65 | 4.13cd | 3.64‒4.62 | 3.77d | 3.38‒4.16 | 4.55bc | 4.24‒4.86 | 5.04ab | 4.72‒5.36 | 5.45a | 5.11‒5.78 | <0.001 |
| C14:0 | 4.71d | 4.14‒5.27 | 6.63b | 5.98‒7.28 | 5.81c | 5.29‒6.32 | 6.54bc | 6.13‒6.95 | 6.96ab | 6.53‒7.39 | 7.55a | 7.10‒8.00 | <0.001 |
| Total MCSFAs | 7.34e | 6.42‒8.27 | 11.64bcd | 10.57‒12.71 | 10.54d | 9.69‒11.39 | 12.15bc | 11.47‒12.82 | 13.11ab | 12.41‒13.81 | 14.08a | 13.35‒14.81 | <0.001 |
| C15:0 | 0.21 | 0.17‒0.24 | 0.21 | 0.16‒0.25 | 0.21 | 0.18‒0.24 | 0.19 | 0.17‒0.22 | 0.20 | 0.17‒0.23 | 0.22 | 0.19‒0.25 | 0.813 |
| C16:0 (PA) | 50.91a | 49.53‒52.29 | 49.77ab | 48.18‒51.36 | 50.72a | 49.46‒51.99 | 49.94ab | 48.94‒50.95 | 48.51ab | 47.46‒49.56 | 48.23b | 47.14‒49.32 | 0.005 |
| C17:0 | 0.38b | 0.33‒0.43 | 0.41ab | 0.35‒0.47 | 0.48a | 0.43‒0.53 | 0.49a | 0.45‒0.53 | 0.48a | 0.44‒0.51 | 0.46ab | 0.42‒0.50 | 0.004 |
| C18:0 | 4.27 | 3.86‒4.68 | 4.48 | 4.01‒4.96 | 4.29 | 3.91‒4.66 | 4.24 | 3.94‒4.54 | 4.34 | 4.02‒4.65 | 4.14 | 3.82‒4.47 | 0.902 |
| C20:0 | 0.20 | 0.17‒0.22 | 0.22 | 0.19‒0.25 | 0.21 | 0.19‒0.23 | 0.21 | 0.19‒0.22 | 0.20 | 0.18‒0.21 | 0.20 | 0.18‒0.22 | 0.741 |
| C22:0 | 0.33 | 0.30‒0.37 | 0.36 | 0.31‒0.40 | 0.32 | 0.28‒0.35 | 0.32 | 0.29‒0.35 | 0.36 | 0.33‒0.39 | 0.34 | 0.31‒0.37 | 0.249 |
| C24:0 | 0.49a | 0.45‒0.53 | 0.37b | 0.32‒0.41 | 0.26cd | 0.23‒0.30 | 0.26c | 0.24‒0.30 | 0.24cd | 0.21‒0.27 | 0.20d | 0.17‒0.23 | <0.001 |
| Total SFAs | 64.12b | 62.85‒65.40 | 67.45a | 65.98‒68.92 | 67.03a | 65.86‒68.20 | 67.81a | 66.88‒68.74 | 67.42a | 66.45‒68.39 | 67.87a | 66.87‒68.88 | 0.002 |
| C14:1n-5 | 0.13 | 0.09‒0.16 | 0.14 | 0.10‒0.18 | 0.15 | 0.12‒0.19 | 0.15 | 0.13‒0.18 | 0.15 | 0.12‒0.17 | 0.14 | 0.11‒0.16 | 0.815 |
| C16:1n-7 | 1.58b | 1.41‒1.75 | 1.71ab | 1.51‒1.91 | 1.99a | 1.84‒2.15 | 1.95a | 1.82‒2.07 | 1.94a | 1.81‒2.07 | 1.92a | 1.79‒2.06 | 0.002 |
| C18:1t | 0.11 | 0.08‒0.14 | 0.09 | 0.05‒0.12 | 0.12 | 0.09‒0.15 | 0.11 | 0.09‒0.13 | 0.09 | 0.06‒0.11 | 0.10 | 0.07‒0.12 | 0.409 |
| C18:1n-9 (OA) | 17.60a | 16.52‒18.68 | 15.08b | 13.84‒16.32 | 14.71b | 13.72‒15.7 | 14.54b | 13.75‒15.32 | 14.56b | 13.74‒15.38 | 14.39b | 13.54‒15.24 | <0.001 |
| C20:1n-9 | 0.51a | 0.46‒0.55 | 0.35b | 0.30‒0.40 | 0.33b | 0.29‒0.37 | 0.31b | 0.27‒0.34 | 0.29b | 0.26‒0.33 | 0.28b | 0.24‒0.31 | <0.001 |
| C22:1n-9 | 0.32a | 0.28‒0.36 | 0.23b | 0.19‒0.28 | 0.20b | 0.17‒0.24 | 0.19bc | 0.16‒0.21 | 0.16bc | 0.13‒0.19 | 0.13c | 0.10‒0.16 | <0.001 |
| C24:1n-9 | 0.51a | 0.47‒0.54 | 0.22b | 0.18‒0.26 | 0.19b | 0.15‒0.22 | 0.20b | 0.17‒0.22 | 0.18b | 0.16‒0.21 | 0.18b | 0.15‒0.20 | <0.001 |
| Total MUFAs | 20.75a | 19.65‒21.85 | 17.82b | 16.55‒19.08 | 17.70b | 16.69‒18.71 | 17.43b | 16.63‒18.24 | 17.36b | 16.53‒18.20 | 17.13b | 16.26‒18.00 | <0.001 |
| C18:2n-6 (LA) | 8.33c | 7.73‒8.94 | 9.36bc | 8.67‒10.06 | 10.63a | 10.08‒11.19 | 10.18ab | 9.74‒10.62 | 10.78a | 10.32‒11.24 | 10.63a | 10.15‒11.11 | <0.001 |
| C18:3n-6 | 0.13b | 0.09‒0.17 | 0.12b | 0.07‒0.17 | 0.19ab | 0.15‒0.23 | 0.22a | 0.19‒0.25 | 0.19ab | 0.16‒0.22 | 0.20ab | 0.16‒0.23 | 0.003 |
| C20:2n-6 | 0.36a | 0.33‒0.38 | 0.20b | 0.17‒0.23 | 0.18b | 0.16‒0.20 | 0.17b | 0.16‒0.19 | 0.16b | 0.14‒0.18 | 0.19b | 0.17‒0.21 | <0.001 |
| C20:3n-6 | 0.35a | 0.32‒0.39 | 0.24b | 0.20‒0.28 | 0.20bc | 0.17‒0.23 | 0.18bc | 0.16‒0.21 | 0.16c | 0.13‒0.19 | 0.15c | 0.12‒0.17 | <0.001 |
| C20:4n-6 (ARA) | 1.39a | 1.32‒1.46 | 0.94b | 0.86‒1.02 | 0.71c | 0.64‒0.77 | 0.71c | 0.65‒0.76 | 0.67c | 0.62‒0.72 | 0.70c | 0.64‒0.75 | <0.001 |
| C22:2n-6 | 0.42a | 0.39‒0.46 | 0.33b | 0.29‒0.37 | 0.28bc | 0.25‒0.31 | 0.24cd | 0.22‒0.27 | 0.22d | 0.19‒0.24 | 0.20d | 0.17‒0.23 | <0.001 |
| C22:4n-6 | 0.96a | 0.91‒1.02 | 0.48b | 0.42‒0.55 | 0.27c | 0.22‒0.32 | 0.28c | 0.23‒0.32 | 0.29c | 0.25‒0.34 | 0.27c | 0.23‒0.32 | <0.001 |
| Total n-6 PUFAs | 11.95 | 11.33‒12.57 | 11.69 | 10.97‒12.40 | 12.46 | 11.89‒13.03 | 11.98 | 11.52‒12.43 | 12.48 | 12.01‒12.95 | 12.33 | 11.84‒12.82 | 0.309 |
| C18:3n-3 (ALA) | 0.63c | 0.51‒0.75 | 0.84bc | 0.70‒0.97 | 1.00ab | 0.89‒1.11 | 1.01ab | 0.92‒1.10 | 1.07a | 0.99‒1.17 | 0.99ab | 0.89‒1.08 | <0.001 |
| C20:5n-3 | 0.11a | 0.09‒0.13 | 0.08ab | 0.06‒0.10 | 0.07b | 0.05‒0.08 | 0.07b | 0.05‒0.08 | 0.07b | 0.06‒0.09 | 0.06b | 0.05‒0.07 | 0.002 |
| C22:5n-3 (DPA) | 0.81a | 0.75‒0.87 | 0.49b | 0.43‒0.56 | 0.41bc | 0.35‒0.46 | 0.39bc | 0.35‒0.43 | 0.38c | 0.33‒0.42 | 0.38c | 0.33‒0.42 | <0.001 |
| C22:6n-3 (DHA) | 1.33a | 1.23‒1.43 | 1.09b | 0.98‒1.20 | 0.84c | 0.75‒0.93 | 0.78c | 0.71‒0.85 | 0.69c | 0.61‒0.76 | 0.70c | 0.63‒0.78 | <0.001 |
| Total n-3 PUFAs | 2.88a | 2.71‒3.04 | 2.50b | 2.30‒2.69 | 2.31bc | 2.15‒2.46 | 2.25bc | 2.12‒2.37 | 2.22bc | 2.09‒2.35 | 2.13c | 1.99‒2.26 | <0.001 |
| Total PUFAs | 14.82 | 14.10‒15.54 | 14.18 | 13.35‒15.01 | 14.76 | 14.10‒15.42 | 14.22 | 13.69‒14.75 | 14.70 | 14.15‒15.25 | 14.46 | 13.89‒15.03 | 0.626 |
| n-6/n-3 | 4.24d | 3.87‒4.61 | 4.82cd | 4.39‒5.25 | 5.54abc | 5.20‒5.89 | 5.58ab | 5.30‒5.85 | 5.76a | 5.47‒6.04 | 6.13a | 5.83‒6.42 | <0.001 |
| LA/ALA | 13.92a | 12.66‒15.19 | 13.23ab | 11.77‒14.69 | 11.81ab | 10.64‒12.97 | 11.48b | 10.55‒12.40 | 10.99b | 10.03‒11.95 | 12.03ab | 11.03‒13.04 | 0.004 |
| ARA/DHA | 1.13 | 1.01‒1.25 | 0.93 | 0.79‒1.06 | 0.91 | 0.80‒1.02 | 1.02 | 0.93‒1.11 | 1.05 | 0.96‒1.14 | 1.10 | 1.01‒1.19 | 0.076 |

Results are presented as mean and 95% confidence interval (CI). Mean values within the same row not sharing a common letter are significantly different (*P*<0.05). MCSFA: medium-chain saturated fatty acid; SFA: saturated fatty acid; MUFA: monounsaturated fatty acid; PUFA: polyunsaturated fatty acid; PA: palmitic acid; OA: oleic acid; LA: linoleic acid; ARA: arachidonic acid; ALA: α-linolenic acid; DPA: docosapentaenoic acid; DHA: docosahexaeonoic acid.
